# Supplementary material for: Substituent Effects of the Nitrogen Heterocycle on Indole and Quinoline HDN Performance: A Combination of Experiments and Theoretical Study
Source: Int J Mol Sci. 2023 Feb 3;24(3):3044. doi: 10.3390/ijms24033044 (PMC9917669; doi:10.3390/ijms24033044)
Supplement: Supplementary file 1 [file ijms-24-03044-s001.zip › ijms-2117152-supplementary.pdf]

1. The chromatograms for indole and Quinoline HDN products and the corresponding positions.

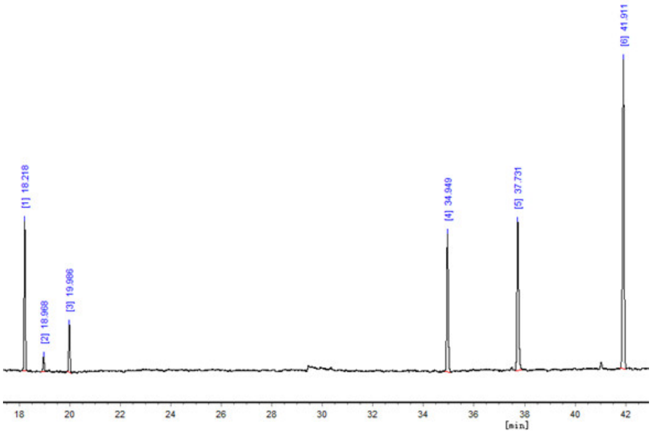

GC-spectrogram of indole HDN product

Peak number	Peak position/min	**Product**	**formula**	**structural**
1	18.2	Ethyl-cyclohexane (ECH)	C_8H_{16}	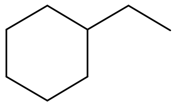
2	18.9	Ethyl-benzene (EB)	C_8H_{10}	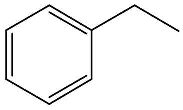
3	20.0	Ethyl-cyclohexene (ECHE)	C_8H_{14}	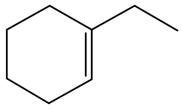
4	34.5	o-ethylaniline (OEA)	C_8NH_{15}	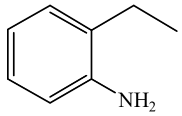
5	37.7	Indoline (DHI)	C_8NH_7	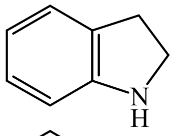
6	41.9	Indole (IND)	C_8NH_5	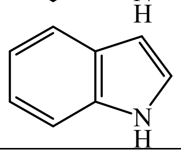

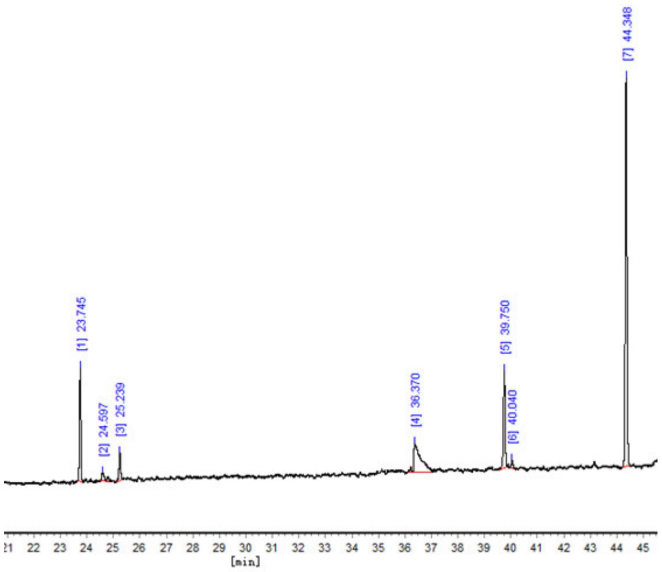

GC-spectrogram of Quinoline HDN product

Peak number	Peak position/min	**Product**	**formula**	**structural**
1	23.8	Propyl-cyclohexane (PCH)	C_9H_{18}	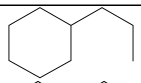
2	24.6	Propyl-benzene (PB)	C_9H_{12}	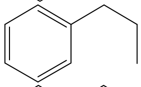
3	25.2	Propyl-cyclohexene (PCHE)	C_9H_{16}	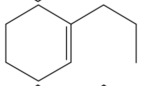
4	37.4	Decahydro-quinoline (DHQ)	C_9NH_{17}	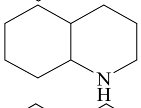
5	39.8	5,6,7,8-tetrahydroquinoline (THQ-5)	C_9NH_{11}	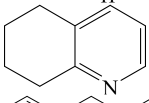
6	40.4	o-propylaniline (OPA)	C_9NH_{13}	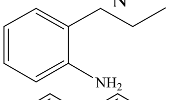
7	43.1	Quinoline (QL)	C_9NH_7	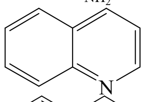
8	44.3	1,2,3,4-tetrahydroquinoline (THQ-1)	C_9NH_{11}	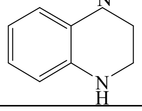

2. HDN products and the corresponding positions of substituent nitrogen compounds

HDN product distribution of 2-methyl-indole (2-M-IND)

Peak number	Peak position/min	**Product**	**formula**	**structural**
1	23.3	iso-Propyl-cyclohexane i-PCH	C_9H_{18}	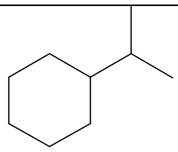
2	23.8	Propyl-cyclohexane (PCH)	C_9H_{18}	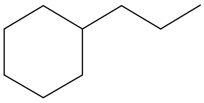
3	25.2	Propyl-cyclohexene (PCHE)	C_9H_{16}	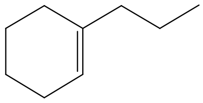
4	39.5	dihydro-2-methyl-indole DH-2-M-IND	C_9NH_{11}	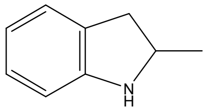
5	40.4	o-propylaniline (OPA)	C_9NH_{13}	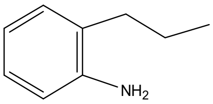
6	44.9	2-M-IND	C_9NH_9	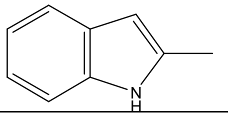

HDN product distribution of 3-methyl-indole (3-M-IND)

Peak number	Peak position/min	**Product**	**formula**	**structural**
1	23.3	i-PCH	C_9H_{18}	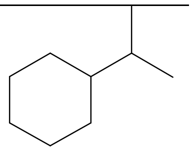
2	24.7	Isopropyl-cyclohexeneyl<br>i-PCHE	C_9H_{16}	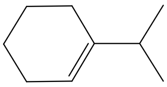
3	26.2	Iso-propenyl-aniline<br>i-OPEA	C_9H_{16}	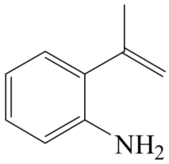
4	39.0	Isopropylaniline<br>i-OPA	C_9NH_{13}	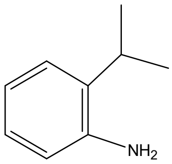
5	41.1	dihydro-3-methyl-indole<br>DH-3-M-IND	C_9NH_{11}	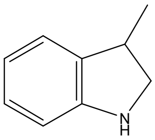
6	45.5	3-M-IND	C_9NH_9	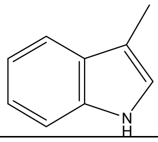

HDN product distribution of 2-methyl-quinoline (2-M-QL)

Peak number	Peak position/min	**Product**	**formula**	**structural**
1	28.1	Butyl-cyclohexane BCH	$C_{10}H_{20}$	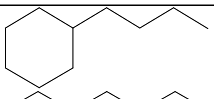
2	29.2	2-butyl-cyclohexene (2-BCHE)	$C_{10}H_{14}$	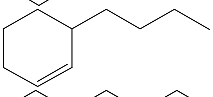
3	29.5	1-butyl-cyclohexene (1-BCHE)	$C_{10}H_{18}$	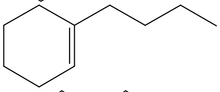
4	37.4	2-methyl-decahydro-quinoline (2-M-DHQ)	$C_{10}NH_{19}$	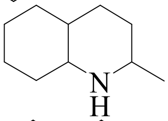
5	42.2	2-methyl-5,6,7,8 tetrahydroquinoline 2-M-THQ-5	$C_{10}NH_{13}$	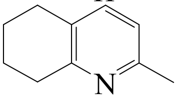
6	44.7	2-M-QL	$C_{10}NH_{9}$	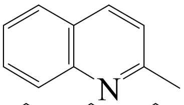
7	45.6	O-butyl-aniline OBA	$C_{10}NH_{15}$	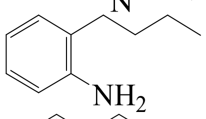
8	46.1	2-methyl-1,2,3,4-tetrahydroquinoline (2-M-THQ-1)	$C_{10}NH_{13}$	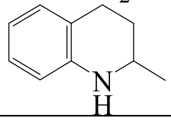

HDN product distribution of 3-methyl-quinoline (3-M-QL)

Peak number	Peak position/min	**Product**	**formula**	**structural**
1	27.6	iso-butyl-cyclohexane<br>i-BCH	$C_{10}H_{20}$	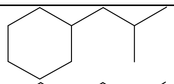
2	28.8	2-iso-butyl-cyclohexene<br>2-i-BCHE	$C_{10}H_{14}$	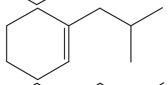
3	29.1	1-iso-butyl-cyclohexene<br>1-i-BCHE	$C_{10}H_{18}$	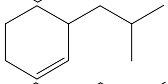
4	38.4	3-methyl-<br>decahydro-quinoline<br>3-M-DHQ	$C_{10}NH_{19}$	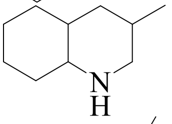
5	44.3	iso-ortho-butyl-aniline<br>i-OBA	$C_{10}NH_{13}$	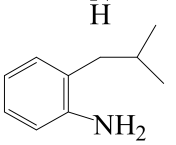
6	44.9	3-methyl-5,6,7,8<br>tetrahydroquinoline<br>3-M-THQ-5	$C_{10}NH_9$	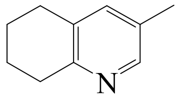
7	46.1	3-M-QL	$C_{10}NH_{15}$	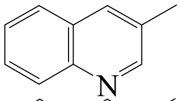
8	47.7	3-methyl-1,2,3,4-<br>tetrahydroquinoline<br>(3-M-THQ-1)	$C_{10}NH_{13}$	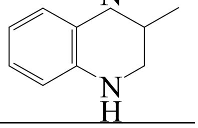
